# Inhibition of WEE1 kinase and cell cycle checkpoint activation sensitizes head and neck cancers to natural killer cell therapies

**DOI:** 10.1186/s40425-018-0374-2

**Published:** 2018-06-21

**Authors:** Jay Friedman, Megan Morisada, Lillian Sun, Ellen C. Moore, Michelle Padget, James W. Hodge, Jeffrey Schlom, Sofia R. Gameiro, Clint T. Allen

**Affiliations:** 1 Translational Tumor Immunology Program, National Institutes on Deafness and Other Communication Disorders, National Institutes of Health, Building 10, Room 7N240C, Bethesda, MD 20892 USA; 2 Laboratory of Tumor Immunology and Biology, National Cancer Institute, National Institutes of Health, Bethesda, MD USA; 3 Department of Otolaryngology-Head and Neck Surgery, Johns Hopkins School of Medicine, Baltimore, MD USA

**Keywords:** NK cells, Resistance, DNA damage checkpoint, WEE1 kinase, haNK cells, KIL cells, Antibody-dependent cell-mediated cytotoxicity

## Abstract

**Background:**

Natural killer (NK) cells recognize and lyse target tumor cells in an MHC-unrestricted fashion and complement antigen- and MHC-restricted killing by T-lymphocytes. NK cells and T-lymphocytes mediate early killing of targets through a common granzyme B-dependent mechanism. Tumor cell resistance to granzyme B and how this alters NK cell killing is not clearly defined.

**Methods:**

Tumor cell sensitivity to cultured murine KIL and human high affinity NK (haNK) cells in the presence or absence of AZD1775, a small molecule inhibitor of WEE1 kinase, was assessed via real time impedance analysis. Mechanisms of enhanced sensitivity to NK lysis were determined and in vivo validation via adoptive transfer of KIL cells into syngeneic mice was performed.

**Results:**

Cultured murine KIL cells lyse murine oral cancer 2 (MOC2) cell targets more efficiently than freshly isolated peripheral murine NK cells. MOC2 sensitivity to granzyme B-dependent KIL cell lysis was enhanced by inhibition of WEE1 kinase, reversing G2/M cell cycle checkpoint activation and resulting in enhanced DNA damage and apoptosis. Treatment of MOC2 tumor-bearing wild-type C57BL/6 mice with AZD1775 and adoptively transferred KIL cells resulted in enhanced tumor growth control and survival over controls or either treatment alone. Validating these findings in human models, WEE1 kinase inhibition sensitized two human head and neck cancer cell lines to direct lysis by haNK cells. Further, WEE1 kinase inhibition sensitized these cell lines to antibody-dependent cell-mediated cytotoxicity when combined with the anti-PD-L1 IgG1 mAb Avelumab.

**Conclusions:**

Tumor cell resistance to granzyme B-induced cell death can be reversed through inhibition of WEE1 kinase as AZD1775 sensitized both murine and human head and neck cancer cells to NK lysis. These data provide the pre-clinical rationale for the combination of small molecules that reverse cell cycle checkpoint activation and NK cellular therapies.

## Background

Natural killer (NK) cells serve an important role in the elimination of malignant cells, in part through recognition of decreased or absent MHC class I expression [1, 2], which is common in many cancer types [3]. Importantly, NK cell recognition and killing of tumor cells is antigen-independent [1]. Thus, NK control of tumor cells complements the antigen-specific, major histocompatibility class (MHC) class I-restricted killing of tumor cells by T-lymphocytes [1]. T-lymphocyte-based cellular therapies induce remarkable responses in subsets of patients with both solid and hematologic malignancies [4, 5], but are limited by MHC-restriction, the expression and presentation of specific antigen, the need for immune-depleting preparative regimens, and treatment logistics. These obstacles may be overcome with NK-based cellular therapies [6].

NK-92 cells are immortalized NK cells derived from a patient with an NK cell lymphoma [7] and have been used as a cell therapy to treat patients with advanced cancer with an acceptable safety profile [8]. NK-92 cells expand in culture and express low levels of the inhibitory receptor killer immunoglobulin-like receptor (KIR), but require exogenous IL-2 for expansion and do not express CD16 required for antibody-dependent cell-mediated cytotoxicity (ADCC). High-affinity NK (haNK cells) are an NK cell therapy product engineered from NK-92 cells to express a CD16 high affinity FcγRIIIa receptor present in 8–14% of the population and produce endogenous IL-2 [9]. haNKs kill carcinoma cells independent of MHC class I expression [9] and can mediate ADCC when combined with IgG1 isotype mAbs [10]. However, incomplete lysis of target cells at 18 h with haNKs alone or in combination with IgG1 mAbs suggests the presence of mechanisms of resistance to NK-mediated killing.

While multiple mechanisms of local immunosuppression within the tumor microenvironment have been identified [11], tumor cell intrinsic mechanisms of resistance to effector immune cell elimination are less well characterized. Seminal work indicates granzyme B, used by both T-lymphocytes and NK cells to kill targets, can results in G2/M cell cycle block [12]. This pause allows time for DNA repair and prevention of mitotic catastrophe and apoptosis [13, 14]. Here, we demonstrated that AZD1775, a small molecule inhibitor of WEE1 kinase, prevented granzyme B-induced G2/M cell cycle checkpoint activation and sensitizes tumor cells to NK killing. Using a newly characterized and culturable murine NK cell line, we showed reversal of cell cycle pause in response to granzyme B through inhibition of CDK1 phosphorylation. This resulted in DNA damage, apoptosis, and enhanced sensitivity to granzyme B-dependent NK lysis of aggressive murine oral carcinoma cells. Treatment of tumor bearing wild-type B6 mice with AZD1775 and adoptive transfer of murine NK cells resulted in enhanced survival over either treatment alone and controls. Similar to results in the syngeneic murine model, treatment of human head and neck cancer cells with AZD1775 sensitized them to both direct haNK lysis and ADCC when combined with the anti-programmed death-ligand 1 (PD-L1) IgG1 mAb Avelumab. These results provide a firm rationale for the clinical combination of NK cell therapy products and targeted therapies that block cell cycle checkpoint activation.

## Methods

### Cell culture

KIL murine NK cells [15] were purchased from Kerafast and cultured in IMDM supplemented with FBS (30% vol/vol), pen/strep, L-glutamine, β-mercaptoethanol, IL-7 (25 ng/mL) and SCF (50 ng/mL) in 6-well plates. KIL cells were maintained at a density of no less than 5 × 10^5^ cells/mL. Four days prior to use in killing experiments, KIL were stimulated with IL-2 (20 ng/mL) to induce proliferation. KIL at low passage number were used for all killing experiments. For some experiments, wild-type C57BL/6 mouse NK cells (WT B6 NK) were isolated via negative magnetic bead separation (Miltenyi) on an autoMACS from splenocytes and used fresh. Mouse oral cancer-2 (MOC2) cells were obtained from Ravindra Uppaluri (Dana-Farber) and maintained in culture as described [16] and used at low passage number. haNK cells obtained from NantBioscience (Culver City, CA) through a cooperative research and drug agreement (CRADA) were cultured in phenol-red free XVIVO media (Lonza) supplemented with 5% human AB serum [9]. For some experiments, human NK cells were isolated via magnetic bead separation from PBMCs obtained from health donor apheresis (NIH Clinical Center Blood Bank, NCT0001846). Isolated human NK cells were cultured overnight in RPMI-1640 supplemented with 10% FCS, pen/strep and L-glutamine overnight before use in assays. UMSCC-6 and -9 cells were obtained from Dr. T.E. Carey (University of Michigan) and used at low passage number [17]. All tumor cell lines were serially verified to be free of mycoplasma or other murine associated pathogens. For some experiments, MOC2 or UMSCC cells were treated with AZD1775 (SelleckChem) reconstituted at high concentration (10 mM) in DMSO. Avelumab was obtained from EMD Serono (a business of Merck KGaA, Darmstadt, Germany) through a CRADA. YAC cells were cultured in RPMI1640 supplemented with FBS (5%). Cell counts were obtained on a Nexcelom Cellometer Auto 2000.

### Flow cytometry

Only fresh cultured cells or tissue prepared into single cell suspensions were analyzed. The harvest and digestion of MOC2 tumors into single cell suspensions for analysis was performed as previously described [18]. Nonspecific surface staining was minimized by staining with CD16/32 (FcR) blocking antibodies for 10 min prior to the addition of primary antibodies when applicable. Anti-mouse NK1.1, CD3, CD16, NKG2D, CD44, CD27, PD-L1, PD-1, Tim-3, CTLA-4, H-2K^b^, H-2D^b^, pan-RAE, H60 and MULT-1 antibodies were from Biolegend. For some experiments, pre-treatment of cells with interferon γ (IFNγ) (20 ng/mL × 24 h) or YAC cells were used as positive controls. Primary antibodies were applied for 30–60 min at concentrations titrated for each antibody. Dead cells were excluded via 7AAD uptake and a “fluorescence-minus-one” technique was used to validate specific staining in all antibody combinations. Intracellular staining was performed with the eBioscience Intracellular Fixation and Permeabilization Buffer Set per manufacturer protocol. Granzyme B and IFNγ antibodies were from Biolegend. All analyses were performed on a BD FACSCanto analyzer running FACSDiva software and interpreted using FlowJo (vX10.0.7r2).

### Indium release assay

KIL cells or sorted WT B6 NK were combined with In^111^ labeled YAC or MOC2 cells at various effector-to-target (E:T) ratios, and KIL or purified NK activity was quantified via In^111^ release at 12 h on a WIZARD2 Automatic Gamma Counter (PerkinElmer).

### Real-time impedance assay

Target MOC2 cells were plated at 5 × 10^3^ cells/well and target UMSCC cells were plated at 1.5 × 10^4^ cells/well. After adherence of target cells overnight, KIL, haNK or isolated NK cells at the indicated E:T ratios were added to target cells plated in a 96-well E-Plate (ACEA Biosciences) and alteration of impedance was acquired using the xCELLigence Real-Time Cell Analysis (RTCA) platform per manufacturer recommendations. For each plot, y-axis is cell index and x-axis is time in hours. Triton X-100 (0.2%) was added to some wells to verify complete loss of cell index with total cell lysis, and effector cells alone were plated up to 1 × 10^6^/well to verify that they do not contribute to gain of impedance (data not shown). In some wells, KIL were exposed to concanamycin A (100 nM) for 4 h, then vigorously washed before being added to target MOC2 cells. Percent loss of cell index for a given time point was calculated as: 1-(experimental cell index/control cell index). All impedance assays were performed in technical triplicate in at least three independent assays to ensure reproducibility.

### Cell cycle analysis

The Click-iT EdU flow cytometry assay kit (Thermo) was used per manufacturer recommendations, with the addition of primary conjugated antibodies for p-HH3^S10^ (clone 11D8) and p-γH2AX^S139^ (clone 2F3) from Biolegend after fixation and permeabilization.

### Western blot analysis

Whole-cell lysates were obtained using NP40 lysis buffer, mixed with NuPAGE LDS sample buffer and NuPAGE sample reducing agent (Life Technologies), heated at 95 °C for 5 min and subjected to electrophoresis using 4–12% Bis-Tris precast gels (Life Technologies) at 150 V for 100 min. The Invitrogen iBlot Dry Blotting System was used to transfer proteins onto a PVDF membrane. Primary antibodies were diluted in 5% BSA prepared from Tween 20-TBS. Anti-mouse CDK1, p-CDK1, p-H2AX and β-actin antibodies were purchased from Cell Signaling Technologies. Blots were incubated with Chemiluminescent HRP Antibody Detection Reagent (Denville Scientific Inc.) and imaged using Image Studio software (LI-COR Biosciences).

### In vivo treatments

All in vivo experiments were approved by the NIDCD Animal Care and Use Committee. WT B6 mice were purchased from Taconic. For tumor growth experiments, MOC2 tumors were established by subcutaneous flank injection of 1 × 10^5^ cells. Treatments were started when tumor reached 0.1 cm^3^ volume, or approximately 14 days after injection. Adoptive transfer of KIL was performed by tail vein injection (TVI) of cells in 200 μL total volume. KIL were irradiated to 18 Gy prior to TVI with a ^137^Cs source (Gammacell-1000) irradiator at a dose rate of 0.74 Gy/min. For some experiments, KIL were labelled with 5 μM carboxyfluorescein succinimidyl ester (CFSE, Sigma Aldrich) as described [19]. AZD1775 used for in vivo experiments was administered via oral gavage in 0.5% methylcellulose. TVI of PBS and oral gavage of 0.5% methylcellulose alone were used as controls. Tumors were measured three times weekly and tumor volume was calculated as: (tumor length x tumor width^2^)/2.

### Statistics

Tests of significance between pairs of data are reported as *p*-values, derived using a Fisher’s exact test or student’s t-test with a two-tailed distribution and calculated at 95% confidence. Comparison of multiple sets of data was achieved with one or two-way analysis of variance (ANOVA). Kaplan-Meier survival curves were compared using the log-rank/Mantel Cox test. Error bars reflect standard deviation from individual experiments. Analysis was performed using GraphPad Prism v6.

## Results

### KIL cells display NK cell surface markers and efficiently lyse target cells

**Fig. 1:**
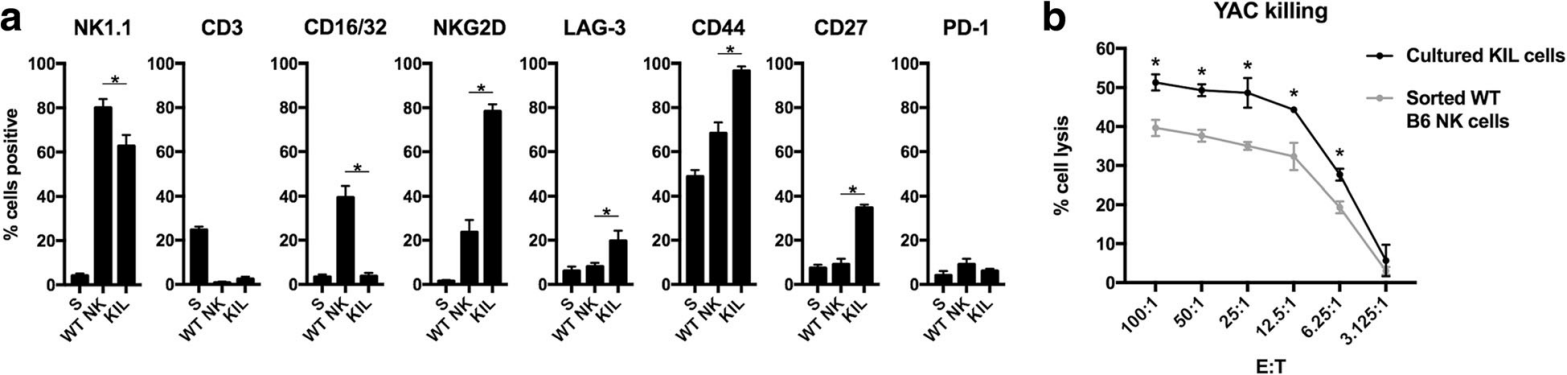
Cultured KIL cells express NK cell surface markers and lyse target cells more efficiently that sorted wild-type NK. **a** KIL cells, sorted WT B6 NK cells and total splenocytes were analyzed for expression of cell surface markers by flow cytometry. Percent cells positive based on isotype staining. Dead cells excluded from analysis via 7AAD positivity. **b** Target (YAC) cell lysis by cultured KIL or sorted WT B6 NK cells at different E:T ratios was assessed via indium release assay (12-h data shown). Three independent assays performed with similar results. * indicates significantly enhanced killing with KIL cells compared to sorted WT B6 NK cells. *, *p* < 0.05

Granzyme B can activate the G2/M cell cycle checkpoint [12]. Inhibition of WEE1 kinase prevents cell cycle checkpoint activation and sensitizes tumor cells to genotoxic and cytotoxic insults [20, 21]. We hypothesized that WEE1 kinase inhibition could sensitize tumor cells to granzyme B-dependent NK killing. To investigate this in a syngeneic model of cancer, we wished to use murine NK cells that can be expanded in culture for in vitro and in vivo experiments. KIL cells are bone marrow-derived killer lymphocytes that maintain NK cell phenotype and function when cultured in IL-7 and SCF [15]. To validate KIL cell phenotype, we explored surface marker expression of these cells compared to freshly sorted WT B6 NK cells. Similar to WT NK cells, KIL cells are NK1.1^+^CD3^−^. KIL express greater levels of the activation markers NKG2D, CD44, CD27 and LAG-3 compared to WT NK (Fig. 1a). Notably, KIL express little CD16/32 (F_c_ receptor), indicating they lack the ability to mediate antibody-dependent cell-mediated cytotoxicity (ADCC). To assess the ability of KIL cells to lyse targets, we compared KIL cell killing of YAC cells to WT B6 NK cells in a standard indium release assay. KIL lysed target YAC cells to a greater degree than WT B6 NK (Fig. 1b). Thus, KIL cells are a culturable cell line that display NK cell surface markers and lyse target cells more efficiently than freshly isolated peripheral murine NK.

### WEE1 kinase inhibition sensitizes oral cancer cells to KIL killing

**Fig. 2:**
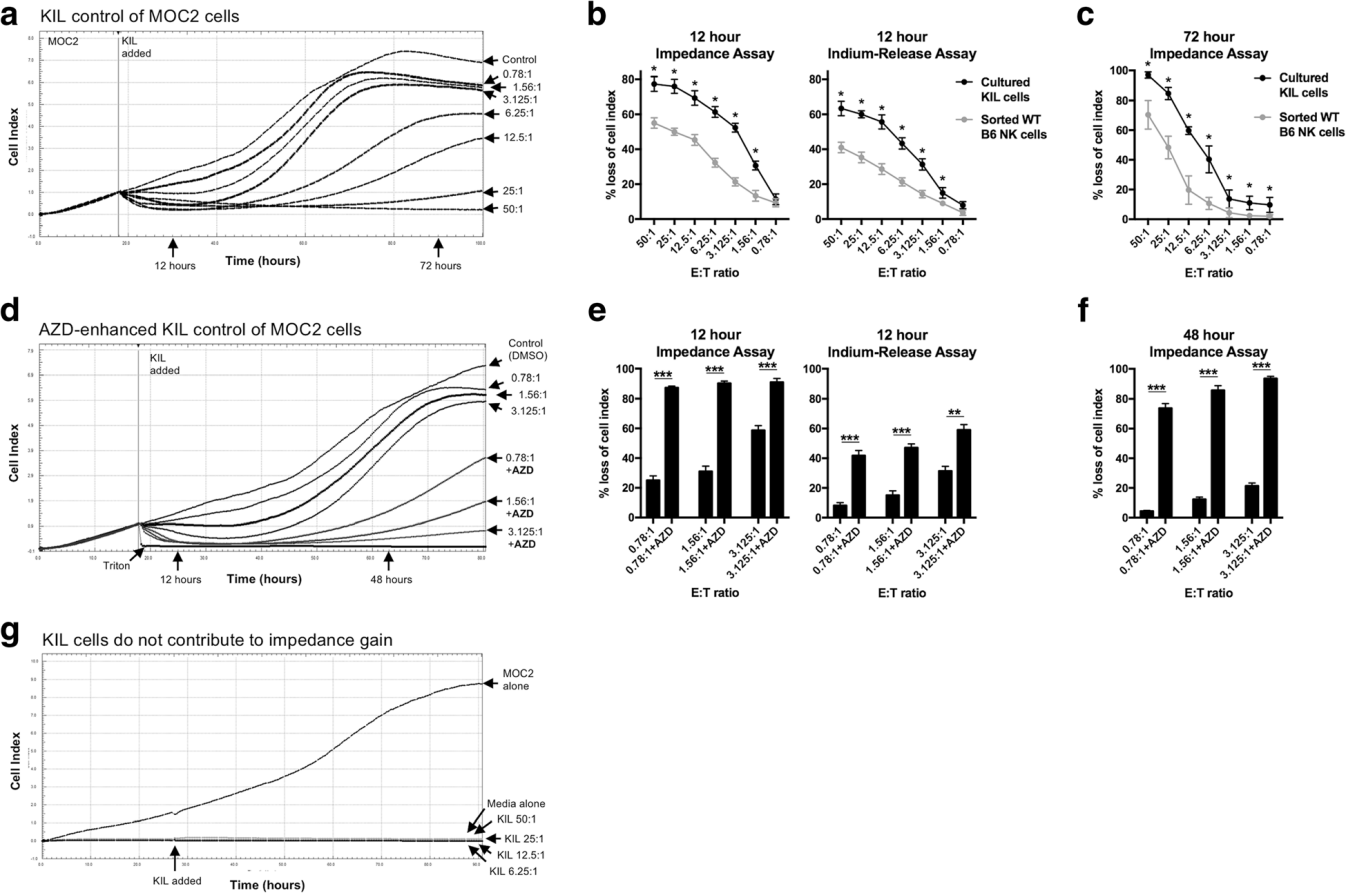
Target cell killing by KIL at low E:T ratios is enhanced following WEE1 kinase inhibition. **a** Loss of cell index of MOC2 oral carcinoma cells following the addition of KIL at increasing E:T ratios was measured via real-time impedance analysis. Vertical line at 18 h indicates time at which KIL were added. Control cells were exposed to KIL media alone. **b** % loss of cell index at 12 h after addition of KIL (black line) or sorted WT B6 NK cells (grey line) quantified on left. On right, for comparison, KIL (black line) or sorted WT B6 NK cells (grey line) were used to induce indium release in a standard 12-h radioactive compound release assay. **c** % loss of cell index at 72 h after addition of KIL (black line) or sorted WT B6 NK cells (grey line) quantified. For B&C, * indicates significantly enhanced killing with KIL cells compared to sorted WT B6 NK cells. **d** Loss of MOC2 cell index following the addition of KIL at low E:T ratios in the presence of AZD1775 (250 nM) or DMSO (volume equivalent). When AZD1775 was present, MOC2 cells were plated in drug at the start of the assay. Maximum loss of cell index was achieved by addition of triton to some wells. % loss of cell index 48 h after the addition of KIL to MOC2 cells is quantified on right. **e** % loss of cell index of MOC2 cells in the presence (AZD1775 250 nM) or absence (DMSO volume equivalent) of WEE1 kinase inhibition 12 h after the addition of KIL at the indicated E:T ratios quantified on left. On right, for comparison, the same was measured in a standard 12-h radioactive compound release assay. **f** % loss of cell index of MOC2 cells 48 h after the addition of KIL. **g** Impedance analysis of MOC2 cells alone (5 × 10^3^ cells/well) compared to media or KIL cells alone up to an E:T ratio equivalent of 50:1 (2.5 × 10^5^ KIL/well). Results presented are representative of three independent experiments with similar results. *, *p* < 0.05; ***, *p* < 0.001

We next assessed the ability of KIL cells to lyse MOC2 target cells. MOC2 cells are carcinogen-induced *Tp53*-mutant (nonsense mutation) oral cavity carcinoma cells that form aggressive, metastatic tumors in vivo that exhibit poor T-lymphocyte infiltration [22–24]. KIL cells induced loss of MOC2 target cell index in a dose-dependent fashion as measured by real-time impedance analysis (Fig. 2a). At low E:T ratios, KIL induced initial loss of cell index compared to DMSO control, but MOC2 cell populations recovered over 24–36 h and escaped growth control. Higher E:T ratios more durably controlled MOC2 growth. Similar assays were performed with WT B6 NK cells. At all E:T ratios 1.56:1 and greater, KIL cells controlled early (12 h) MOC2 cell growth to a greater degree than WT B6 NK (Fig. 2b, left panel). For direct comparison and to validate the impedance platform, we performed standard 12-h indium release cytotoxicity assays using both KIL cells and WT B6 NK cells and effectors and MOC2 cells as targets. Similar to the impedance analysis, KIL cells induced more MOC2 cytotoxicity compared to WT B6 NK cells (Fig. 2b, right panel). One advantage of real-time impedance analysis is longer duration assays that are not possible with radioactive compound release assays. After 72 h, KIL cells controlled MOC2 cell growth to a greater degree than WT B6 NK (Fig. 2c).

MOC2 cells demonstrated resistance to low, physiologic E:T ratios of KIL cells with early growth rebound. However, when target MOC2 cells were plated in the WEE1 kinase inhibitor AZD1775 at low nanomolar (250 nM) concentrations at or below trough levels based on human pharmacokinetics [25], KIL growth control of MOC2 cells at low E:T ratios was significantly enhanced at both early and late time points (Fig. 2d). To again validate the impedance platform, early 12-h growth control measured by change in cell index was directly compared to a standard 12-h indium release cytotoxicity assay with similar trends (Fig. 2e, left and right panels). AZD1775-enhanced KIL control of MOC2 cells was durable with significantly enhanced loss of MOC2 cells index at 72 h (Fig. 2f). Changes in cell index are a direct measure of MOC2 cell adherence, as KIL cells do not contribute to impedance (Fig. 2g). Cumulatively, WEE1 kinase inhibition with AZD1775 sensitizes MOC2 cells to KIL cell-mediated cytotoxicity at low E:T ratios.

### MOC2 cells are sensitized to KIL killing through a tumor cell-dependent mechanism

**Fig. 3:**
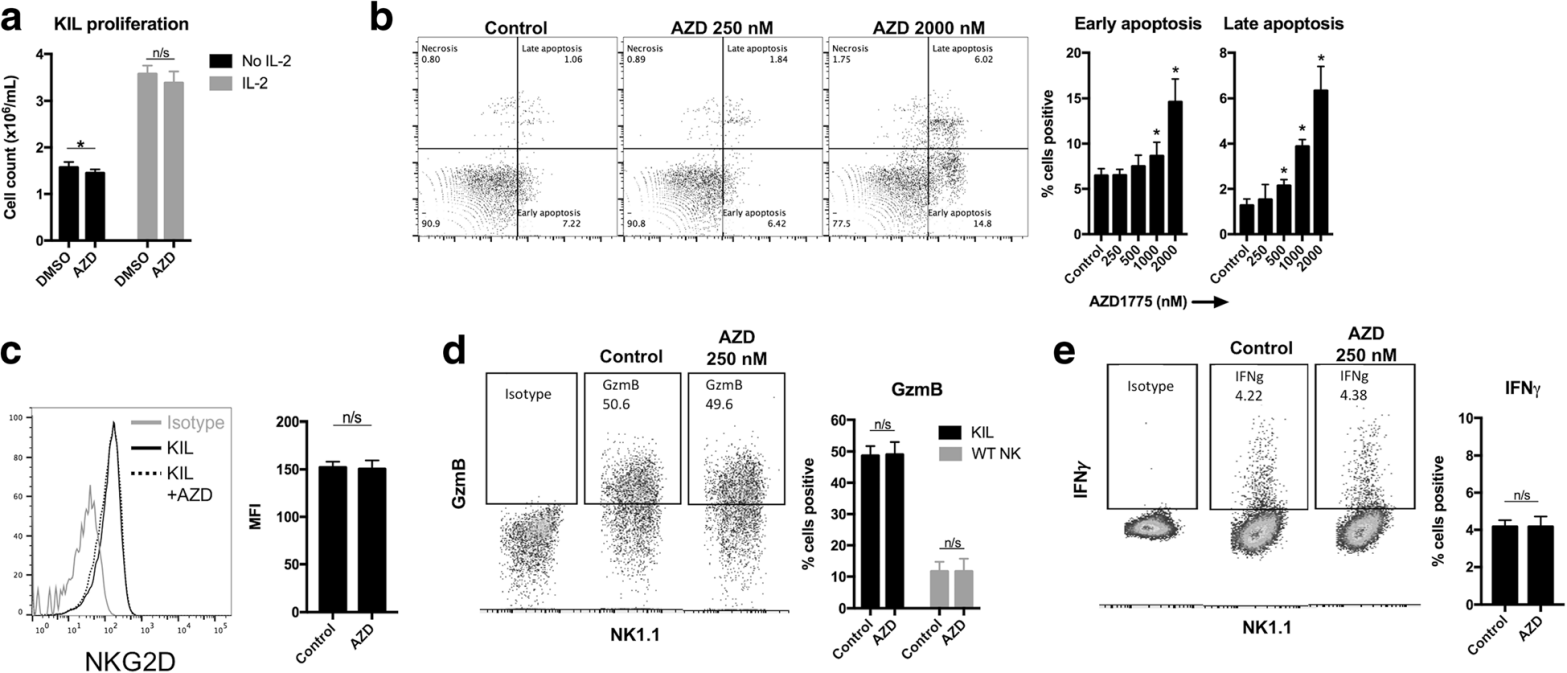
WEE1 kinase inhibition does not significantly alter KIL proliferation, viability or production of cytotoxic compounds. **a** KIL cells were cultured in AZD1775 (250 nM) or DMSO volume equivalent for 48 h in the presence or absence of IL-2 (20 ng/mL) and cell counts were obtained. **b** KIL cells were cultured in increasing doses of AZD1775 (250–2000 nM) or DMSO volume equivalent for 18 h and apoptosis was assessed by annexin V/7AAD flow cytometry. Representative dot plots shown on left, quantified for early (annexin V^+^7AAD^−^) or late (annexin V^+^7AAD^+^) apoptosis on right. * indicates significant difference compared to control. **c** KIL cells were cultured in AZD1775 (250 nM) or DMSO for 18 h and analyzed for expression of NKG2D by flow cytometry. Representative overlaid histograms on left, quantified on right. **d** KIL or sorted WT NK cells (stimulated with 20 ng/mL IL-2) were cultured in AZD1775 (250 nM) or DMSO for 18 h and analyzed for granzyme B accumulation via intracellular flow cytometry. Representative positive and isotype control dot plots of KIL cells shown on left, with KIL and sorted WT B6 NK staining quantified on the right. **e** KIL cells were combined with MOC2 cells at an E:T ratio of 3:1 for 4 h in the presence of brefeldin A, then KIL cells were assayed for IFNγ accumulation by intracellular flow cytometry. Representative positive and isotype control dot plots shown on left, quantified on the right. Results presented are representative of three independent experiments with similar results. *, *p* < 0.05. n/s, not significant

To explore possible mechanisms of AZD1775 enhanced lysis of MOC2 targets by KIL cells, we first ruled out significant changes in direct KIL cell function or viability upon exposure to drug. AZD1775 at 250 nM did not alter IL-2 induce proliferation of KIL cells (Fig. 3a), and induction of KIL apoptosis in the presence of AZD1775 was only observed at concentrations greater than 250 nM (Fig. 3b). AZD1775 250 nM did not alter expression of the activating NK cell receptor NFG2D on KIL cells (Fig. 3c). AZD1775 250 nM did not alter intracellular granzyme B levels of cultured KIL cells or WT B6 NK cells stimulated with IL-2 (Fig. 3d). Lastly, upon exposure to target MOC2 cells, AZD1775 250 nM did not significantly modulate IFNγ production by KIL cells (Fig. 3e).

**Fig. 4:**
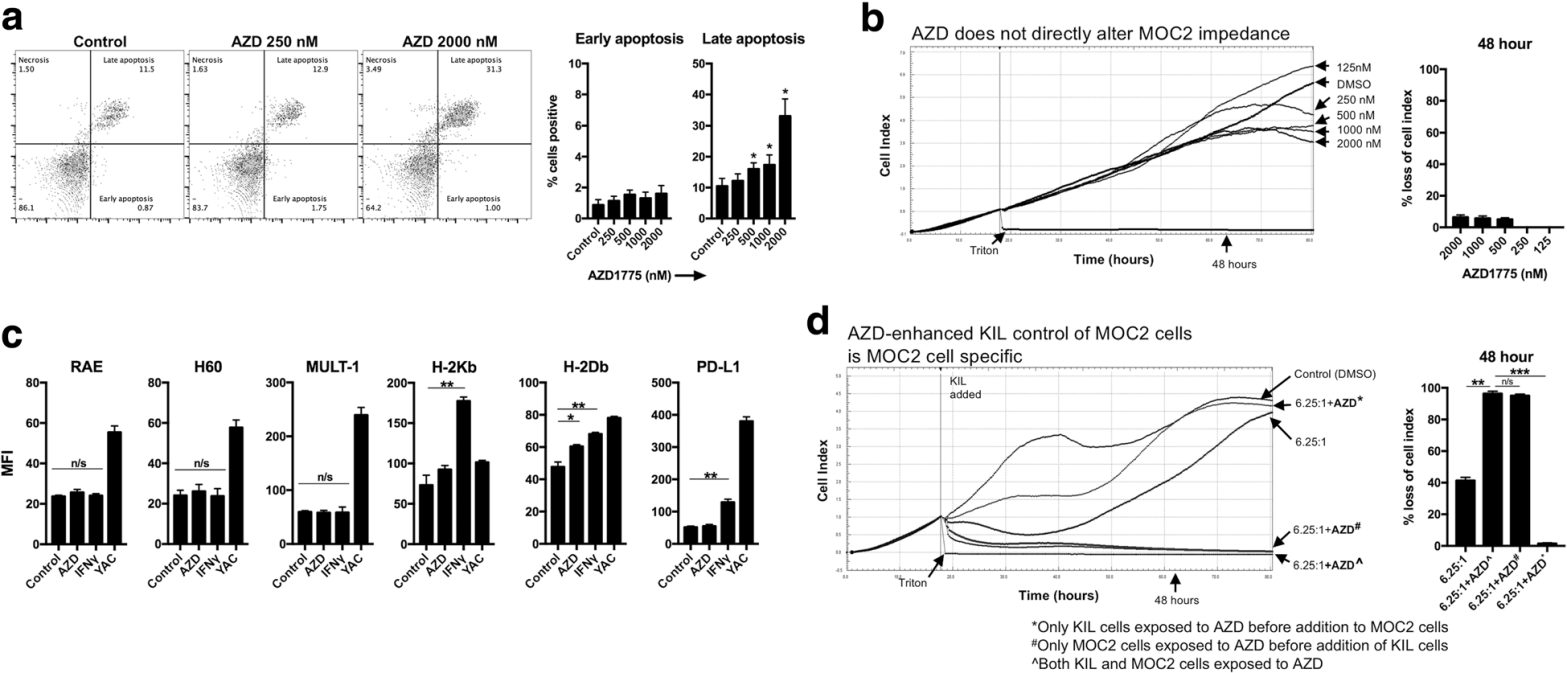
WEE1 kinase inhibition sensitizes MOC2 cells to NK killing via a tumor-specific mechanism. **a** MOC2 cells were cultured in increasing doses of AZD1775 (250–2000 nM) or DMSO volume equivalent for 18 h and apoptosis was assessed by annexin V/7AAD flow cytometry. Representative dot plots shown on left, quantified for early (annexin V^+^7AAD^−^) or late (annexin V^+^7AAD^+^) apoptosis on right. * indicates significant difference compared to control. **b** MOC2 cells were plated in increasing concentrations of AZD1775 (125–2000 nM) or DMSO volume equivalent and assays for cell proliferation and viability via impedance analysis, % loss of cell index at 48 h quantified on right. **c** MOC2 cells were exposed to AZD1775 (250 nM) or DMSO (control, volume equivalent) for 18 h and analyzed for cell surface marker expression by flow cytometry. IFNγ (20 ng/mL × 18 h) treatment of MOC2 cells or YAC cells were used as positive controls. **d** MOC2 cells were plated in AZD1775 250 (nM) or DMSO and cell index after exposure to KIL at a 6.25:1 E:T ratio was assessed by impedance analysis. In some wells, MOC2 cells were plated in AZD1775 (250 nM) or DMSO but washed three times before the addition of KIL (only MOC cells exposed to AZD before addition of KIL cells). In other wells, KIL were exposed to AZD1775 or DMSO for 24 h, then washed three times before being added to MOC2 cells (only KIL cells exposed to AZD before addition to MOC2 cells). % loss of cell index 48 h after the addition of KIL to MOC2 cells quantified on the right. Results presented are representative of three independent experiments with similar results. *, *p* < 0.05; **, *p* < 0.01; ***, *p* < 0.001; n/s, not significant

We next assayed for changes in target MOC2 cells in response to WEE1 kinase inhibition. AZD1775 250 nM alone did not induce MOC2 cell apoptosis, while dramatic dose-dependent apoptosis was induced at greater concentrations (Fig. 4a). As measured by impedance analysis, AZD1775 alone at concentrations up to 2000 nM did not induce significant loss of MOC2 cell index through 48 h (Fig. 4b). We next assessed MOC2 cells for changes in cell surface expression of MHC class I, PD-L1 and NKG2D ligands in the presence of AZD1775250 nM. AZD1775 modestly induced H-2D^b^ expression but had no significant effects on H-2K^b^, PD-L1 or NKG2D ligands RAE, H60 and MULT-1 (Fig. 4c), suggesting that alterations in the expression of cell surface determinants of NK sensitivity could not explain enhanced KIL sensitivity. We next selectively exposed either target MOC2 cells alone or KIL cells alone to AZD1775 250 nM before assessing MOC2 sensitivity to KIL cell lysis (Fig. 4d). Selective exposure of MOC2 cells, but not KIL cells, to AZD1775 enhanced MOC2 susceptibility to KIL-induced loss of cell index. Taken together, these data indicate that AZD1775 enhances KIL growth control of MOC2 via a tumor cell-dependent mechanism, but is not due to direct MOC2 cytotoxicity or altered expression of MHC class I, PD-L1 or NKG2D ligands.

### WEE1 kinase inhibition reverses tumor cell G2/M cell cycle checkpoint activation following granzyme B exposure leading to enhanced susceptibility to KIL killing

**Fig. 5:**
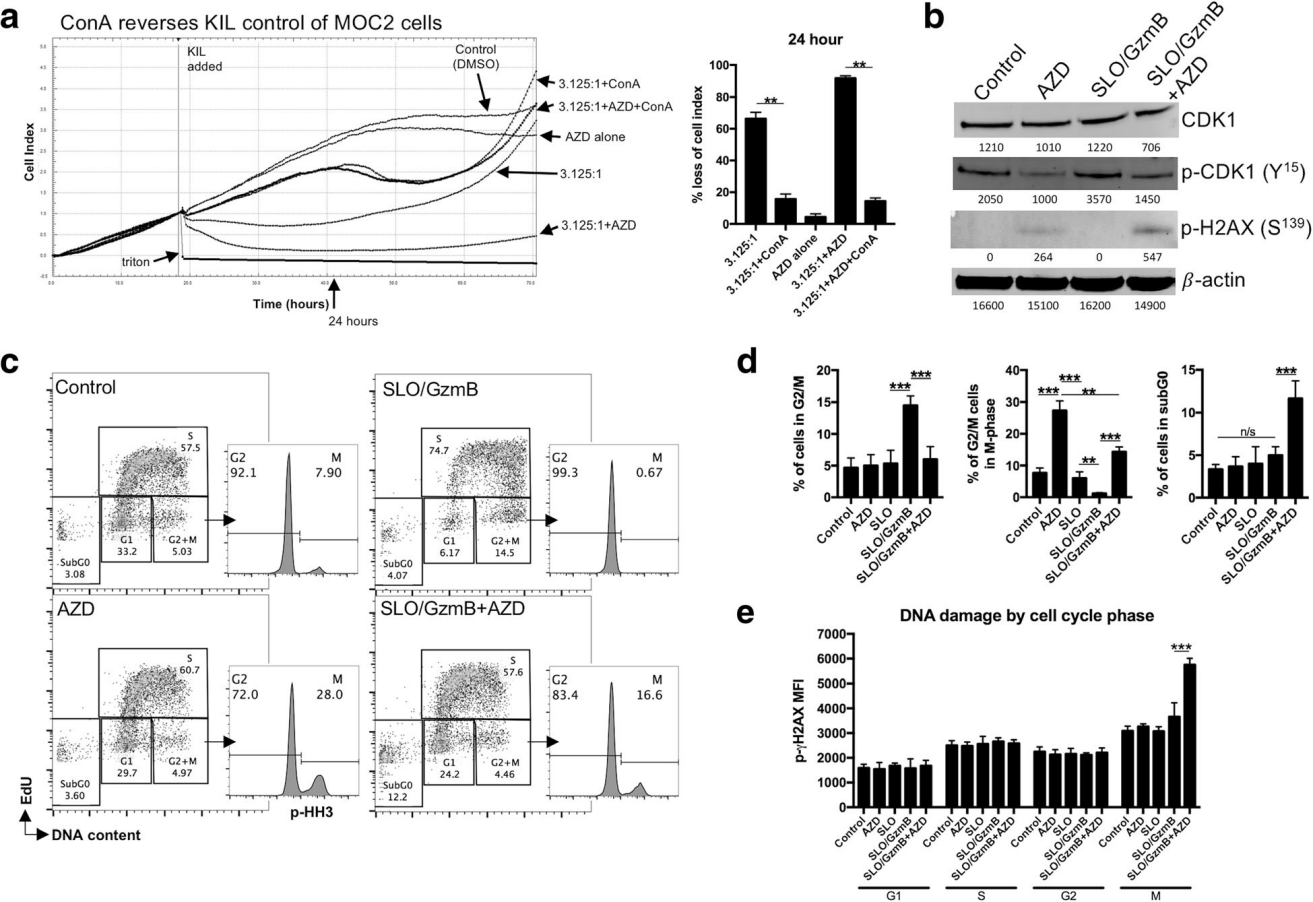
WEE1 kinase inhibition reverses granzyme B-induced MOC2 G2/M accumulation resulting in DNA damage, DNA fragmentation and cell death. **a** MOC2 cells were plated in AZD1775 250 (nM) or DMSO and cell index after exposure to KIL at a 3.125:1 E:T ratio was assessed by impedance analysis. In some wells, KIL were exposed to concanamycin A (100 nM) for 4 h then washed three times before addition to MOC2 cells. % loss of cell index 24 h after the addition of KIL to MOC2 cells quantified on right. **b** Western blot analysis of total CDK1, p-CDK1^Y15^ and p-H2AX^S139^ after treatment of MOC2 cells as in B. Densitometry listed under each blot. Results presented are representative of three independent experiments with similar results. **c** MOC2 cells were exposed to AZD1775 (250 nM), streptolysin O (40 ng/mL)/Granzyme B (1.5 μg/mL) or the combination for 2 h, then analyzed for cell cycle distribution via 4D cell cycle flow analysis (DNA content, EdU uptake, p-HH3^S10^ staining and p-H2AX^S139^ staining). DMSO (volume equivalent) used for control. Representative dot plots for each treatment condition shown with histogram inserts indicating cells in mitosis (p-HH3^S10^ positive). **d** Quantification of cells within select phases of the cell cycle. **e** Cell cycle phase-dependent quantification of DNA damage, as measured by p-H2AX^S139^ staining, for each treatment condition. *, *p* < 0.05; **, *p* < 0.01; ***, *p* < 0.001

We next assessed KIL cell control of MOC2 cells in the presence or absence of AZD1775 following pre-treatment of KIL with concanamycin A (ConA). ConA blocks perforin activity that is required for granzyme B-induced cell death. Perforin inhibition significantly reversed both baseline and AZD1775-enhanced KIL control of MOC2 tumor cells (Fig. 5a), suggesting that AZD1775 enhanced granzyme B-dependent control of MOC2. To investigate on-target effects of AZD1775, we performed western blot analysis of CDK1 phosphorylation, the target of WEE1 kinase (Fig. 5b). Recombinant granzyme B, combined with streptolysin O to allow entry into the cytoplasm of target cells, induced phosphorylation of CDK1. Baseline and granzyme B-induced CDK1 phosphorylation was reduced with AZD1775. As CDK1 phosphorylation was reversed, phosphorylation of γH2AX increased with significant induction in the combination treated cells. Multi-parameter cell cycle analysis revealed that granzyme B induced a G2/M cell cycle block (Fig. 5c), with accumulation of MOC2 cells in the G2/M gate but very few cells demonstrating phosphorylation of histone H3 (HH3), a mitosis marker. AZD1775 alone and in combination pushed cells through this G2/M cell cycle checkpoint with M-phase accumulation. Combination granzyme B and AZD1775 resulted in significant accumulation of cells in the subG0 phase of the cell cycle, indicative of DNA fragmentation and apoptosis (quantified in Fig. 5d). Quantitative measurement of DNA damage by p-γH2AX^S139^ flow cytometry revealed that accumulation of DNA damage occurred primarily within M-phase cells, with significant induction of DNA damage following treatment with combination granzyme B and AZD1775 (Fig. 5e). These data suggest that AZD1775-induced sensitization of MOC2 cells to KIL occurs at least through reversal of granzyme B-induced G2/M cell cycle checkpoint activation, leading to significant M-phase DNA damage and induction of apoptosis.

### WEE1 kinase inhibition sensitizes oral cavity tumors to growth control by adoptively transferred KIL in vivo

**Fig. 6:**
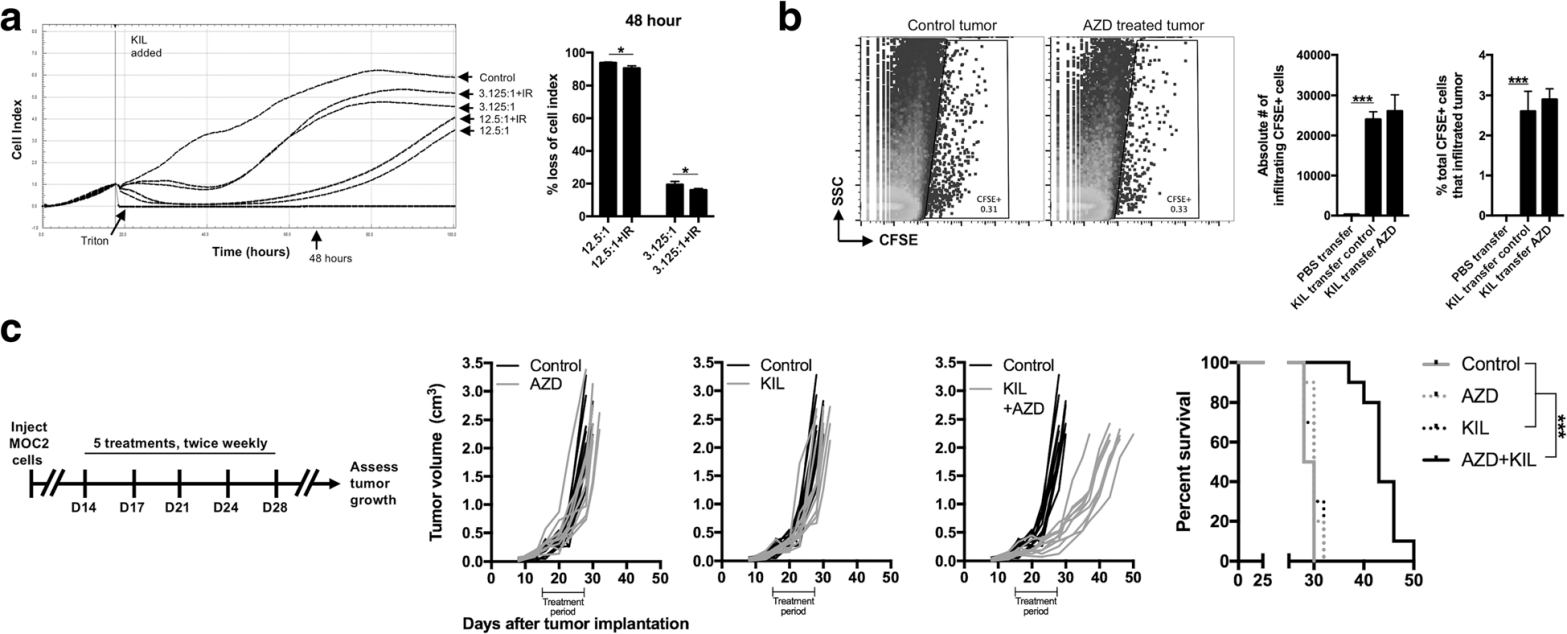
WEE1 kinase inhibition sensitizes MOC2 tumors to control by adoptively transferred KIL cells. **a** KIL cells were irradiated to 18 Gy and assessed for control of MOC2 cell index via impedance analysis at E:T ratios of 12.5:1 and 3.125:1. % loss of cell index 48 h after the addition of control or irradiated KIL to MOC2 cells is quantified on the right. **b** KIL cells were CFSE labelled, then adoptively transferred (tail vein injection) into MOC2 tumor-bearing WT B6 mice following 2 doses of AZD1775 (120 mg/kg via oral gavage) or control. Eight hours after tail vein injection of 1 × 10^7^ KIL or PBS, mice (*n* = 5/group) were euthanized and MOC2 tumors were harvested, processed into a single cell suspension, and assessed for CFSE-positive cells via flow cytometry. Quantification of CFSE-labelled cell entry into AZD treated or untreated MOC2 tumors quantified on right. **c** Treatment schema and in vivo treatment of MOC2 tumor-bearing WT B6 mice (*n* = 10 mice/group) with adoptively transferred KIL (1 × 10^7^ cells via tail vein injection) alone or in combination with AZD1775 (120 mg/kg via oral gavage). For each of five treatments with AZD1775 and KIL, AZD1775 was administered 8 h before adoptive transfer of KIL. Control mice were treated with oral gavage of carrier only and tail vein injection of PBS only. Individual tumor growth curves shown for each cohort, with survival on the right. Experiment was repeated in two independent assays with similar results. *, *p* < 0.05; ***, *p* < 0.001

To validate our in vitro findings in vivo, we adoptively transferred KIL cells into MOC2 tumor-bearing WT B6 mice. To mimic human NK products, we irradiated KIL (18 Gy) before adoptive transfer. This irradiation did not significantly reduce the ability of KIL cells to control MOC2 growth (Fig. 6a). Additionally, we adoptively transferred CFSE-labeled KIL cells into control and AZD1775-treated mice bearing MOC2 tumors and used flow cytometry to verify entry of KIL cells into the MOC2 tumor microenvironment (Fig. 6b). Treatment of mice with AZD1775 did not enhance KIL infiltration into MOC2 tumors. While AZD1775 or adoptively transferred KIL cells alone had little impact on MOC2 primary tumor growth, the combination delayed MOC2 primary tumor growth and significantly prolonged tumor-bearing mouse survival (Fig. 6c).

### WEE1 kinase inhibition sensitizes human head and neck cancer cells to both direct NK lysis and antibody-dependent cell-mediated cytotoxicity

**Fig. 7:**
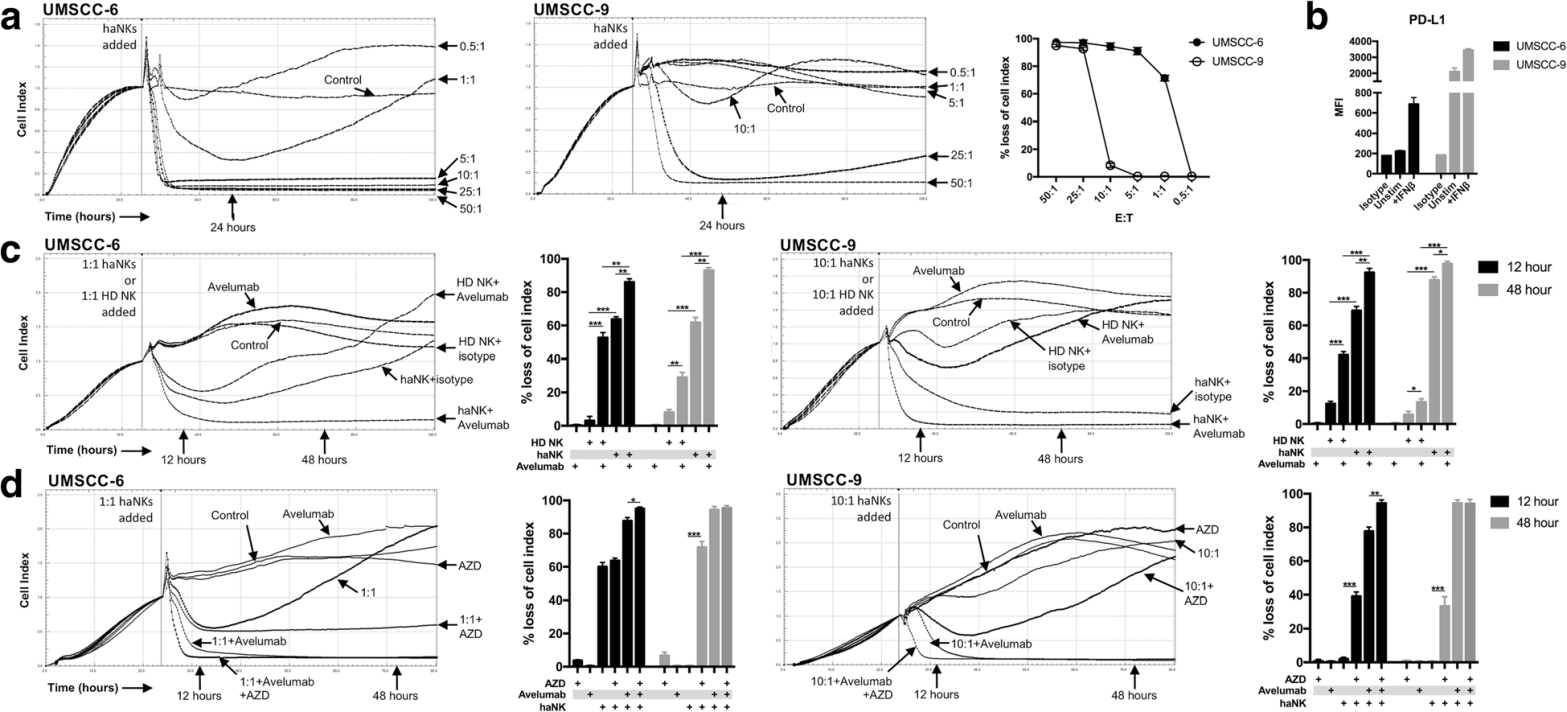
WEE1 kinase inhibition sensitizes human head and neck cancer cells to direct and ADCC-mediated NK cell lysis. **a** Loss of cell index of UMSCC-6 or − 9 cells following the addition of haNK cells at increasing E:T ratios was measured via real-time impedance analysis. Vertical line at ~ 24 h indicates time at which haNKs were added. Control cells were exposed to haNK media alone. % loss of cell index 24 h after the addition of haNKs is quantified on right. **b** UMSCC-6 and -9 cells were assessed for cell surface PD-L1 expression by flow cytometry in the presence of absence of IFN stimulation (10 μg/mL × 24 hours). **c** Loss of cell index following the addition of haNKs or healthy donor (HD) NK cells to UMSCC-6 (at 1:1 E:T ratio) or − 9 (10:1 E:T ratio) treated with Avelumab (2 ng/mL) or isotype control was measured via real-time impedance analysis. Where indicated, Avelumab was added at the same time haNK or HD NK cells were added. % loss of cell index 12 and 48 h after the addition of haNKs is quantified to the right of each plot. **d** Loss of cell index following the addition of haNKs to UMSCC-6 (at 1:1 E:T ratio) or − 9 (10:1 E:T ratio) treated with Avelumab or isotype control and plated in the presence or absence of AZD1775 (AZD) at 250 nM was measured via real-time impedance analysis. % loss of cell index 12 and 48 h after the addition of haNKs is quantified to the right of each plot. All in vitro experiments were repeated in at least three independent assays with similar results. *, *p* < 0.05; **, *p* < 0.01; ***, *p* < 0.001

Hypothesizing that we could replicate our murine findings in human SCC cells, we measured the ability of human haNKs [9] to induce loss of cell index of UMSCC-6 (HPV^−^ base of tongue carcinoma) or − 9 (HPV^−^ oral tongue carcinoma) cells via impedance analysis. haNKs induced dose-dependent loss of cell index when added to both UMSCC-6 and -9 cells (Fig. 7a). After validating baseline and IFN-inducible expression of PD-L1 on both UMSCC cell lines (Fig. 7b), we next assessed whether haNKs were capable of mediating ADCC when combined with Avelumab, an IgG1 monoclonal antibody targeting PD-L1. Consistent with results from Jochems, et al. [10], the addition of Avelumab significantly enhanced haNK control of UMSCC-6 cells at a 1:1 E:T ratio and UMSCC-9 cells at a 10:1 E:T ratio (Fig. 7c). The addition of an antibody blocking the CD16 receptor attenuated loss of cell index induced by haNK plus Avelumab treatment beyond that observed with haNK cells alone (data not shown), verifying this enhanced loss of cell index was mediated through ADCC. Further, in a head-to-head comparison, the ability of haNKs to induce both direct and Avelumab-ADCC mediated loss of cell index was significantly greater than heathy donor NK cells in both UMSCC cell lines. We next assessed whether the addition of AZD1775 at 250 nM altered direct or Avelumab-ADCC mediated haNK control of UMSCC cells. After exposure of UMSCC-6 cells to haNKs at a 1:1 E:T ratio and UMSCC-9 cells to haNKs at a 10:1 E:T ratio with or without Avelumab, AZD175 exposure significant enhanced early (12 h) and durable long-term (48 h) loss of cell index in both models (Fig. 7d). These data suggest that, similar to murine MOC2 oral cancer cells, WEE1 kinase inhibition sensitizes human head and neck cancer cells to direct and ADCC mediated NK killing.

## Discussion

These studies are important for several reasons. First, we further characterized and validated the murine KIL NK cell line [15] that can be used for pre-clinical studies of NK cell biology. The C57BL/6 genetic background of these cells makes them useful in a wide array of syngeneic pre-clinical models. Next, using KIL cells as a murine model of an NK cell therapeutic, we mechanistically demonstrated that an inhibitor of WEE1 kinase, AZD1775, can be used to reverse tumor cell G2/M cell cycle checkpoint activation (leading to a G2/M block) following exposure to NK cell granzyme B, leading to increased tumor cell DNA damage and cell death. AZD1775 sensitized murine MOC2 carcinoma cells to granzyme B-dependent KIL lysis, and treatment of immunocompetent MOC2-tumor bearing B6 mice with the combination of AZD1775 and adoptively transferred KIL cells enhanced survival over either treatment alone. We also provided further evidence, in addition to that from Jochems et al. [9], that human haNK cells efficiently lyse HPV^−^ HNSCC cells, both via direct lysis and Avelumab-mediated ADCC. Finally, we validated that WEE1 kinase inhibition can sensitize human HNSCC cancer cells to both direct and ADCC-mediated NK lysis. These data provide a pre-clinical rationale for the combination of WEE1 kinase inhibitors, or other inhibitors of DNA cell cycle checkpoint activation, with NK cellular therapies.

Following exposure to cytotoxic/genotoxic insults such chemotherapy and ionizing radiation, cancer cells activate cycle checkpoints leading to cell cycle pause [20, 21]. This allows time for DNA damage repair and accumulation of nucleosides required for such repair. Cancer cells lacking functional TP53 rely exclusively on the G2/M cell cycle checkpoint for such cell cycle pauses as TP53 serves as the master regulatory of the G1/S cell cycle checkpoint [20, 21, 26, 27]. WEE1 kinase serves as a master regulator of the G2/M cell cycle checkpoint [28]. Seminal work by Greenberg et al. demonstrated that constitutive activity of WEE1 kinase blocks granzyme B-induce apoptosis [12]. Here, we demonstrate cell cycle block at G2/M following exposure of cells to granzyme B, and reversal of this block with AZD1775 leading to DNA damage and apoptosis. Accumulation of DNA damage within M-phase cells after exposure to AZD1775 is consistent with induction of mitotic catastrophe as a mechanism of cell death and apoptosis [13, 14]. Exposure of MOC2 cells to granzyme B alone did not induce measureable DNA damage, suggesting that CDK1 phosphorylation and G2/M cell cycle pause after granzyme B is not just a simple DNA damage response. The formal mechanism(s) behind granzyme B-induced CDK1 phosphorylation and cell cycle pause are being investigated.

Recent work from our laboratory demonstrated similar cell cycle responses in a number of syngeneic tumor cell lines upon exposure to T-lymphocytes (Sun et al. *Cancer Immunol Res*, In Press). Here, we demonstrated that WEE1 kinase inhibition also sensitized murine and human cancer cells to granzyme B-dependent NK killing independent of MHC class I, PD-L1 or NKG2D ligand expression [29, 30], suggesting that inhibition of cell cycle checkpoint activation may be a more universal approach to sensitizing tumor cells to immunotherapy. As with any systemic treatment, the net effects on both tumor cells and immune cell function and survival must be considered. While AZD1775 exposure to KIL cells alone did appear to modestly blunt their ability to lyse MOC2 target cells, it had little impact on KIL expansion in the presence of IL-2 or viability and the net effect following exposure of both tumor and murine KIL or human haNK cells to AZD1775 was enhanced tumor cell susceptibility to NK killing.

MOC2 cells harbor few genomic alterations, are highly aggressive, have low baseline and IFN-inducible MHC class I expression. They form tumors in mice that demonstrate very poor T-lymphocyte infiltration [22, 31]. T-lymphocyte based immunotherapies such as synthetic STING agonists [18], PD- or CTLA-4-based immune checkpoint inhibitors [24, 32] and MDSC suppressing treatments [24] have no effect on the survival of mice bearing MOC2 tumors. That MOC2 cells appear sensitive to KIL lysis at low E:T ratios is evidence supporting that tumor cells with IFN response defects or low antigenicity can be targeted with NK cellular therapies. Yet, human NK-based cell therapies have not garnered attention similar to T-lymphocyte-based cellular therapies. There are practical reasons for this; infusions with activated, allogeneic NK cells may cause acute graft-versus-host disease due to T-lymphocyte alloreactivity [33] and supply of autologous or allogeneic NK cells is limited as NK cells represent 2% or less of circulating white blood cells [6].

These obstacles may be overcome by haNK cells. Engineered from the NK-92 immortalized lymphoma cell line to have the capacity to mediate ADCC through expression of a CD16 high affinity FcγRIIIa receptor and produce endogenous IL-2 [9, 10], haNKs expand readily in culture and represent an “off the shelf” NK cellular therapeutic that is currently in clinical trials to treat advanced solid malignancies (NCT03027128). Importantly, haNKs can mediate ADCC when combined with humanized or chimeric IgG1 isotype mAbs. Here, following evaluation of an underlying mechanism and in vivo validation of enhanced sensitivity to NK killing following WEE1 kinase inhibition with our murine model, we demonstrated that AZD1775 sensitized human cancer cells to both direct and ADCC-mediated killing. Furthermore, our use of the PD-L1 mAb Avelumab for ADCC brings up the possibility that this mAb therapeutic could both enhance T-lymphocyte function through PD-blockade and mediate NK-based ADCC. Carefully executed clinical trials combining haNK adoptive transfer and Avelumab would be needed to investigate this possibility.

## Conclusions

Using a newly characterized and culturable murine NK cell line, we demonstrated that WEE1 kinase inhibition can be used to prevent G2/M cell cycle checkpoint activation and sensitize tumor cells to granzyme B-dependent NK lysis. Concurrent treatment of adoptively transferred murine NK cells with WEE1 kinase inhibition in immunocompetent mice bearing aggressive tumors prolonged survival over either treatment alone. Inhibition of WEE1 kinase sensitized human cancer cells to both direct granzyme B-dependent haNK killing and ADCC when combined with the IgG1 mAb Avelumab. The WEE1 kinase inhibitor AZD1775, Avelumab and haNKs are all in various stages of clinical development and have demonstrated acceptable safety profiles. Combined with a mechanism-based rationale for combination, these data strongly support the clinical evaluation of NK-based cell therapies, with or without an IgG1 mAb to mediate ADCC, in combination with agents that inhibit cell cycle checkpoint activation.

## Abbreviations

ADCC: Antibody-dependent cell-mediated cytotoxicity
CDK1: Cyclin-dependent kinase 1
CTL: Cytotoxic T-lymphocyte
haNK: High affinity NK
IFN: Interferon
KIR: Killer immunoglobulin-like receptor
MHC: Major histocompatibility complex
MOC2: Mouse oral cancer 2
NK: Natural killer
PD-L1: Programmed death-ligand 1
